# Impact of boost irradiation on pelvic lymph node control in patients with cervical cancer

**DOI:** 10.1093/jrr/rrt097

**Published:** 2013-08-02

**Authors:** Masaru Wakatsuki, Tatsuya Ohno, Shingo Kato, Ken Ando, Shin-ei Noda, Hiroki Kiyohara, Kei Shibuya, Kumiko Karasawa, Tadashi Kamada, Takashi Nakano

**Affiliations:** 1 Research Center for Charged Particle Therapy, National Institute of Radiological Sciences, 4-9-1 Anagawa, Inage-ku, Chiba 263-8555, Japan; 2 Department of Radiation Oncology, Gunma University Graduate School of Medicine, 3-39-22 Showa-machi, Maebashi, Gunma 371-8511, Japan; 3 Department of Radiation Oncology, Saitama Medical University International Medical Center, 1397-1 Yamane, Hidaka, Saitama 350-1298, Japan

**Keywords:** uterine cervical cancer, radiation therapy, boost irradiation, lymph node metastasis, pelvic lymph node

## Abstract

Radiation therapy (RT) for metastatic pelvic lymph nodes (PLNs) is not well established in cervical cancer. In this study the correlation between size of lymph nodes and control doses of RT was analyzed. Between January 2002 and December 2007, 245 patients with squamous cell carcinoma of the cervix treated with a combination of external beam irradiation with or without boost irradiation and high-dose rate brachytherapy were investigated. Size of lymph node was measured by computed tomography before RT and just after 50 Gy RT. Of the 245 patients, 78 had PLN metastases, and a total of 129 had enlarged PLNs diagnosed as metastases; 22 patients had PLN failure. The PLN control rate at 5 years was 79.5% for positive cases and 95.8% for negative cases. In cases with positive PLNs, 12 of 129 nodes (9.3%) developed recurrences. There was significant correlation between PLN control rate and size of PLN after 50 Gy (<10 mm: 96.7%, ≥ 10 mm: 75.7 % (*P*<0.001)). In addition, the recurrence in these poor-response nodes was significantly correlated with dose of RT. Nine of 16 nodes receiving ≤ 58 Gy had recurrence, but none of 21 nodes receiving > 58 Gy had recurrence (*P* = 0.0003). These results suggested that the response of lymph nodes after RT was a more significant predictive factor for recurrence than size of lymph node before RT, and poor-response lymph nodes might require boost irradiation at a total dose of > 58 Gy.

## INTRODUCTION

Although success in using radiation therapy (RT) for cervical cancer with concurrent cisplatin-based chemotherapy has recently been demonstrated, the 5-year survival rate for advanced cervical cancer is still around 55% [1–5], and the treatment for locally advanced tumors is in need of even more aggressive therapy. Strategies for novel treatment of locally advanced tumors include use of other drugs in the form of radiosensitizing chemotherapy [6–8], increasing the irradiation dose to improve the effect of the brachytherapy [9–11], and use of particle therapy [12]. These methods are expected to achieve better outcomes for locally advanced cervical cancer.

Several researchers have reported that 39–44% of patients with advanced cervical cancer have pelvic lymph node (PLN) metastases (based on lymphadenectomy) [13–15]. However, as many lymph node metastases cannot be detected by computed tomography (CT) or magnetic resonance imaging (MRI), RT for locally advanced cervical cancer includes prophylactic irradiation to the PLN area as standard radiation therapy for advanced cervical cancer.

However, irradiation to enlarged PLNs is not well established as standard therapy in the case of detectable lymph node metastases on MRI and CT. It is assumed that control of enlarged lymph nodes will be of clinical significance in patients who do not have local failure. In many facilities, boost irradiation has been used only empirically on positive PLNs. In several clinical trials, the treatment for patients with PLN metastases has not been adequately reported [16–18], and the relationship between irradiation dose and tumor control is still unclear. Hence the treatment for PLN metastases needs to be further developed and refined so as to improve the treatment for advanced cervical cancer. The purpose of the current study was to analyze the relationship between PLN control, irradiation dose and PLN size.

## MATERIALS AND METHODS

### Patient characteristics

Between January 2002 and December 2007, at the Department of Radiation Oncology, Gunma University, and at the National Institute of Radiological Sciences, 245 patients newly diagnosed with cervical cancer were reviewed. They met the following criteria: International Federation of Gynecology and Obstetrics (FIGO) clinical Stage Ib1–IVa, histologically squamous cell carcinoma, curative treatment intent, and treatment of external irradiation with high dose-rate brachytherapy.

Patients were initially evaluated by their medical history, physical examination, and routine hematologic and serum chemistry laboratory studies. All patients underwent imaging with CT of the chest, abdomen and pelvis, and MRI of the pelvis, before RT and just after 50 Gy. Clinical stage was determined according to the FIGO classification [19].

CT images were interpreted in routine clinical fashion. Lymph node status was classified by short axis on CT images as negative (<1 cm) or positive (≥1 cm). For positive lymph nodes, both long and short axes were recorded to the nearest millimeter.

### Radiotherapy

Patients were treated with a combination of external beam irradiation and high-dose-rate (HDR) intracavitary brachytherapy or interstitial brachytherapy. External irradiation was delivered with 10 MV photons by using antero–posterior and postero–anterior parallel opposing ports. The common whole pelvic field borders were at the interspace of the L4–5 vertebrae superiorly, at the inferior border of the obturator foramen inferiorly, and at 1.5–2 cm lateral to the bony pelvis. After the start of the brachytherapy, a central shield was used in whole pelvic fields, i.e. lower two-thirds of pelvis in the case of N1. The fraction for external irradiation was mostly 1.8–2 Gy midplane tumor dose daily, with four to five fractions weekly to the pelvic lesion. Doses to the whole pelvic fields ranged from 20–39.6 Gy (median 30.6 Gy), and doses to the total pelvis, consisting of the combined doses to the whole pelvic and the central shielding fields, ranged from 25.2–51 Gy (median 50.6 Gy). When the pelvic lymph node region was treated, the boosted field treatment was done via two or four ports, and the doses ranged from 6–10 Gy, which each radiation oncologist decided case by case. Treatment planning CT was performed once again for boost RT planning in almost all cases, but the first planning CT was reused in some cases. We calculated and used the biologically equivalent dose of 2-Gy fractions (EQD2) according to the linear quadratic model for incomplete sublethal damage repair [20].

### Brachytherapy

After whole pelvic irradiation, intracavitary brachytherapy by a remote after-loading system using an iridium-192 source was performed. Three to five fractions (median: four fractions) were administered once per week at a fraction dose of 5–7 Gy at Point A, with the total dose ranging from 18–30 Gy (median 24 Gy). In the analysis of the dose to lymph node, the dose of brachytherapy was not considered because image-guided brachytherapy was not done in many of the cases. This schedule of external beam and brachytherapy irradiation is the standard schedule in Japan [21]. For cases of bulky tumor, interstitial brachytherapy was performed as previously described [10].

### Chemotherapy

A total of 89 patients received concurrent chemotherapy, consisting of five weekly cycles of cisplatin (40 mg/m^2^). The other 156 patients were treated with irradiation alone.

### Lymph node evaluation and follow-up

After completion of radiotherapy, patients were followed up every 1–3 months for 2 years, and every 3 or 6 months thereafter. The examination consisted of a physical examination, routine blood cell counts, chemistry profile, chest X-ray, and CT scan of the chest, abdomen and pelvis. Suspected persistent or recurrent disease was confirmed by biopsy whenever possible. Treatment failures were classified as local recurrences, pelvic lymph node recurrences or distant metastases.

### Statistical analysis

Time to recurrence was measured from the date of the start of treatment. The Kaplan–Meier method was used to derive estimates of overall survival and lymph node control. Tests of the equivalence of the estimates of overall survival and lymph node control rates were performed using the log-rank statistic. Chi-square test (Yates' correction) was used for statistical analysis of the correlation between lymph node control and clinical characteristics, and between clinical stages and lymph node metastases. The Mann–Whitney U-test was used for statistical analysis of the correlation between lymph node control and lymph node size or lymph node external irradiation dose.

## RESULTS

The patient age ranged from 30 to 88 years, with a mean of 63 years. The numbers of patients with Stage I, II, III and IV disease were 44, 87, 93 and 21, respectively. Of the 245 patients, 18 had para-aortic lymph node metastases. Staging laparotomy was not performed, and no histologic confirmation of CT-positive pelvic or para-aortic lymph nodes was obtained. No patient underwent lymph node resection. The median follow-up period for surviving patients and all patients was 62 months and 55 months, respectively.

Of the 245 patients, 78 (31.8%) had enlarged PLNs. Clinical stages of the cases with enlarged PLNs were I: 2 of 44, II: 16 of 87, III: 47 of 93, and IV: 13 of 21. The clinical stages of the cases with enlarged PLNs were more progressive than those of the cases without enlarged PLNs (*P*<0.001). In the cases of enlarged PLNs, 129 lymph nodes were detected in the pelvis. Size of lymph nodes on CT image, location of lymph nodes, and total dose of external radiation for lymph nodes are shown in Table 1.

**Table 1.** Characteristics of lymph node status

**PLN-positive case**			
Number/case	1–5	mean :1.7	
		median: 1	
Size (short axis)	10–36 mm	mean: 13.1 mm	
		median: 12 mm	
Size (long axis)	10–40 mm	mean: 16.6 mm	
		median: 14 mm	
Location	External iliac:82	Internal iliac: 14	Common iliac: 19 Obturator: 14
SDose of external irradiation	45–60.6 Gy	mean: 55.4 Gy	Boost : 46 cases, 76 nodes
		median: 56 Gy	Non-boost: 32 cases, 53 nodes
Dose of EQD2	43.2–60.8 Gy	mean: 55.0 Gy	
		median: 56 Gy	

**Fig. 1.**
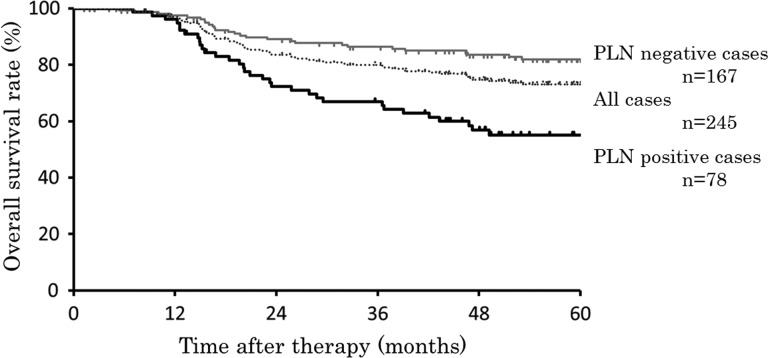
Overall survivals of all, PLN-positive, and PLN-negative cases. Dotted line is that of all cases (*n* = 245), black line is that of PLN-positive cases (*n* = 78), and gray line is that of PLN-negative cases (*n* = 167).

**Fig. 2.**
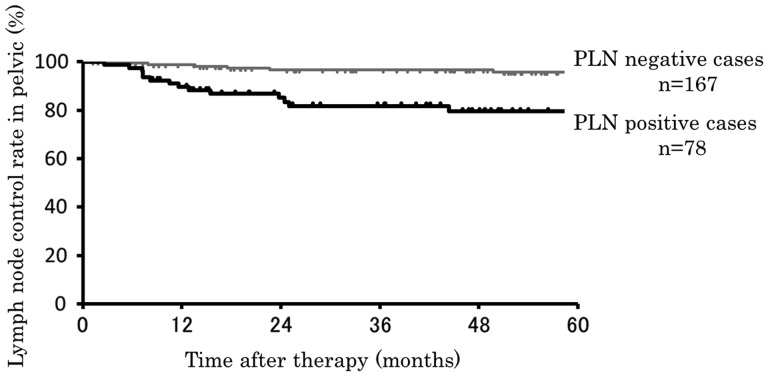
Pelvic lymph node control of PLN-positive and PLN-negative cases. Black line is that of PLN-positive cases (*n* = 78), and gray line is that of PLN-negative cases (*n* = 167).

The overall survival of cases by PLN status is shown in Figure 1. Five-year overall survival rates of all cases, PLN-positive and PLN-negative cases were 73.1%, 55.1% and 82.0%, respectively. There were significant differences for overall survival between PLN-positive and -negative cases (*P*<0.001). Of the 245 patients, 20 (8.2%) showed recurrence into the PLNs. Five-year PLN control rates in cases with positive and negative PLNs were 79.5% and 95.8%, respectively (*P*<0.001; Figure 2). Of the 245 patients, 35 had local failure, and 14 of these 35 patients had LN recurrence. In the cases without enlarged PLNs, 6 of 167 cases (3.6%) showed PLN recurrence. In the cases with enlarged PLNs, 14 of 78 cases (17.9%) showed PLN recurrence, with five of them having PLN recurrence in lymph nodes different from those before RT.

Analyzing the respective lymph nodes, 117 of 129 nodes (90.7%) were controlled. The correlation between lymph node control and clinical characteristics, such as FIGO staging, boost therapy, chemotherapy, para-aortic lymph node metastases and serum levels of squamous cell carcinoma antigen, are shown in Table 2; there were no significant correlations.

**Table 2.** The correlation between lymph node control and clinical characteristics, such as FIGO staging, boost therapy, chemotherapy, para-aortic lymph node metastases and serum levels of squamous cell carcinoma antigen

		**PLN recurrence**			
		+	−	**Total**	***P*-value**
**FIGO**	1	**0**	**2**	2	0.414
	2	**4**	**12**	16	
	3	**6**	**41**	47	
	4	**4**	**9**	13	
**Boost**	+	**12**	**34**	46	0.052
	−	**2**	**30**	32	
**Number of lymph nodes**	1	**5**	**37**	42	0.228
	≥2	**9**	**27**	36	
**Chemotherapy**	+	**12**	**39**	51	0.146
	−	**2**	**25**	27	
**Para-aortic lymph node metastases**	+	**6**	**12**	18	0.112
	−	**8**	**52**	60	
**SCC (ng/ml)**	<10	**4**	**29**	33	0.346
	≥10	**10**	**33**	43	

Table 3 shows the correlation between lymph node recurrence and size of lymph node (long and short axes) before RT, and after 50 Gy, or external radiation dose. There were no significant differences between lymph node recurrence and control in size of lymph node (long and short axes) before RT or dose of external radiation (*P* = 0.174, *P* = 0.246 and *P* = 0.496, respectively), but there were significant differences in both long and short axes after 50 Gy (*P* = 0.003 and *P* = 0.004, respectively).

**Table 3.** The correlation between lymph node recurrence and size of lymph node before RT, after 50 Gy, or external radiation dose

	Lymph nodes of recurrence after RT *n* = 12	Lymph nodes of control by RT *n* = 117	*P-*value
Long axis before RT (mm)	12–30 Mean 18.9, Median 17	10–40 Mean 16.3, Median 14	0.174
Short axis before RT (mm)	10–22 Mean 14.5, Median 12	12–36 Mean 12.9, Median 12	0.246
Long axis after 50 Gy (mm)	1–25 Mean 12.8, Median 10	1–36 Mean 7.5, Median 7	0.003
Short axis after 50 Gy (mm)	1–26 Mean 9.8, Median 10	1–26 Mean 5.9, Median 6	0.004
EQD2 (Gy)	50–60 Mean 55.9, Median 56	43.2–60.8 Mean 54.9, Median 56	0.496

When these lymph nodes were divided into a good response group (<10 mm in long axis after 50 Gy) and a poor response group (≥10 mm in long axis after 50 Gy), there was significant correlation between lymph node recurrence and response of lymph node after 50 Gy (*P* = 0.001). Of the 92 nodes, 89 (96.7%) were controlled in the good response group. The total dose of external radiation in the good response group ranged from 48.3–60.8 Gy (mean, 54.1 Gy; median, 50 Gy). On the other hand, 9 of 37 nodes (24.3%) were recurrent in the poor response group. The total dose of external radiation in the poor response group ranged from 43.2–60.2 Gy (mean, 57.3 Gy; median, 59.4 Gy). Furthermore, the correlations between lymph node recurrence and dose of external radiation were analyzed in the good response and poor response groups (Table 4). There was no significant correlation between lymph node recurrence and dose of external radiation in the good response group, but there was significant correlation between those in the poor response group. Nine of 16 nodes (56.3%) receiving ≤ 58 Gy were recurrent, but none of 21 nodes receiving > 58 Gy were recurrent (*P* = 0.0003).

**Table 4.** The correlations between lymph node recurrence and dose of external radiation were analyzed in the good response and poor response groups

	Long axis after 50 Gy ≥ 10 mm (poor response group)			Long axis after 50 Gy <10 mm (good response group)		
	Rec	Control	Total	Rec	Control	Total
≤58 Gy (EQD2)	**9**	**7**	**16**	**1**	**58**	**59**
>58 Gy (EQD2)	**0**	**21**	**21**	**2**	**31**	**33**
		***P* = 0.0003**			***P* = 0.604**	
Total	**9**	**28**	**37**	**3**	**89**	**92**
		***P* = 0.001**				

Rec = recurrence.

## DISCUSSION

In the current study, there were significant differences in size of lymph node after 50 Gy between lymph node recurrence and control, but there were no significant differences in size of lymph node before RT or external radiation dose between them. When these lymph nodes were divided into a good response group and a poor response group, 89 of 92 (96.7%) and 25 of 37 (75.7%) lymph nodes were controlled by RT, respectively. There was a significant correlation between lymph node response and PLN control. On the other hand, neither size of lymph node before RT nor dose of external radiation showed significant correlations with PLN control. Our results suggested that the response of lymph nodes to RT was a more significant predictive factor for PLN control than size of lymph node before RT or dose of external radiation.

In several reports on lymphadenectomy for advanced cervical cancer, 39–45 % patients had PLN metastases [13–15]. In the current study, only 31.8% patients had PLN metastases based on our criteria. It is possible that that the PLN-negative patients might have had latent metastases. Grigsby *et al*., in their study on lymph node control in cervical cancer, reported that three of 165 patients without enlarged PLNs had PLN recurrences [22]. In the present study, six of 167 (3.6%) patients without PLN metastasis had PLN recurrences. However these six patients had local failures before PLN recurrences. Hence these PLN recurrences might be secondary lymph node metastases as a result of local failure. Although the correlation between these PLN recurrences and the irradiation doses is unclear, most latent lymph node metastases that are<10 mm in diameter on CT imaging might be controllable by the current irradiation without boost.

Boost irradiation to PLN metastases has not yet been established as standard therapy. In this study, 78 of 245 patients had 129 lymph nodes in the pelvis, and 117 of them (90.7%) were controlled. Of the 92, 89 (96.7%) nodes were controlled with or without boost in the good response group (Table 4). Only three nodes in two patients were recurrences. One patient did not have local tumor control and the other had multiple lymph node metastases before PLN recurrence. Hence these recurrences might have been secondary metastases. The mean and median doses of external irradiation for these nodes were 54.9 Gy and 50 Gy, respectively. In addition, 58 of 59 lymph nodes were controlled with ≤ 58 Gy. Grigsby *et al*. showed that 2 of 43 patients with CT lymph nodes >1 cm had lymph node recurrences. Their mean lymph node dose was 67.2 Gy [22]. This dose was the combined external irradiation dose and intracavitary brachytherapy dose (details were not shown). In our study, the lymph node doses by brachytherapy were not analyzed because image-guided brachytherapy was not done in many of the cases. Lee *et al*. reported the lymph node doses for HDR brachytherapy [23]. In their study, average doses for the lymph nodes ranged from 3.45–5.45 Gy per four fractions. Hence the mean dose with the combined external irradiation dose and intracavitary brachytherapy dose was<60 Gy. However, Grigsby *et al*. reported that 33 patients with para-aortic metastases received 45 Gy of external irradiation and that no patients had para-aortic lymph node failure [22]. Thus, it was concluded that good response lymph nodes in the pelvis could be controlled by<60 Gy of irradiation from combined external and brachytherapy irradiation.

In the current study, 9 of 37 (24.3%) lymph nodes with poor response to RT were recurrences. When these lymph nodes were divided into two groups by the total dose of external radiation, 9 of 16 (56.3%) were recurrences in the nodes receiving ≤ 58 Gy, but all nodes were controlled in the nodes receiving > 58 Gy. There was a significant correlation between lymph node recurrence and dose of external radiation in the poor response group (*P* = 0.0003). These results suggest that poor response lymph nodes need boost irradiation, at least, for a total dose of > 58 Gy. There were no Grade 3 or greater toxicities, and no correlation between dose of external radiation and late morbidity in these patients, but high-dose boost irradiation to bulky lymph nodes may have the risk of colon and small intestine high-dose exposure because of their close proximity to pelvic lymph nodes. The use of high-precision radiation therapy such as image-guided stereotactic body radiation therapy or particle radiotherapy is expected to be beneficial for such patients. Choi *et al*. reported image-guided stereotactic body radiation therapy for para-aortic lymph node metastases [24], and Kato *et al*. reported carbon ion radiotherapy for uterine cervical cancer [12]. By using these recent technologies, higher doses can be delivered to the tumor without increasing doses to normal tissue. In the future, these new methods might be improved for their application in the treatment of cervical cancer with PLN metastases.

Concurrent chemotherapy is expected to be another strategy for the treatment of PLN metastases. In this study, there were no significant correlations between PLN failure and chemotherapy. However, several researchers have tested new drugs for radiosensitizing chemotherapy, such as concurrent cisplatin/paclitaxel or cisplatin/gemcitabine chemoradiation for locally advanced cervical cancer [6, 7]. In addition, several molecular targeting agents are being used for cancer patients. The combination treatment of RT and these new drugs will be expected to improve the treatment for PLN metastasis in the near future.

In the present study, the diagnosis of lymph node metastasis by MRI and CT had the limitation of being an image-based diagnosis without biopsy. The sensitivity of CT and MRI for PLN metastasis was 57.5% and 55.5%, respectively [25]. Therefore, false-positive lymph nodes might be included in cases of lymph node metastasis. Recently, several researchers reported the effectiveness of PET and MRI diffusion-weighted imaging (MRI-DWI) for lymph node metastasis. The sensitivities of PET and MRI-DWI were 74.7% and 84.6%, respectively [25, 26]. It is thought important that the diagnosis of lymph node metastasis can be expected to improve using the combination of CT, MRI, MRI-DWI and PET.

## CONCLUSION

We analyzed the predictability of PLN control by RT in patients with cervical cancer. There were significant correlations of lymph node control with size of lymph node after 50 Gy. Good-response lymph nodes might not need more than 58 Gy of external irradiation with boost, and poor-response lymph nodes need boost irradiation at a total dose of over 58 Gy.

## FUNDING

This work was partially supported by Grant-in-Aid for Young Scientists B for Japan Society for the Promotion of Science Grant Number 24791354.
